# Structural variant calling: the long and the short of it

**DOI:** 10.1186/s13059-019-1828-7

**Published:** 2019-11-20

**Authors:** Medhat Mahmoud, Nastassia Gobet, Diana Ivette Cruz-Dávalos, Ninon Mounier, Christophe Dessimoz, Fritz J. Sedlazeck

**Affiliations:** 1 Human Genome Sequencing Center, Baylor College of Medicine, Houston, USA; 2 Center for Integrative Genomics, University of Lausanne, Lausanne, Switzerland; 3 Swiss Institute of Bioinformatics, Lausanne, Switzerland; 4 Department of Computational Biology, University of Lausanne, Lausanne, Switzerland; 5 University Center for Primary Care and Public Health, Lausanne, Switzerland; 6 Centre for Life’s Origins and Evolution, Department of Genetics, Evolution & Environment, University College London, London, UK; 7 Department of Computer Science, University College London, London, UK

**Keywords:** Structural variant (SV) detection, De novo assembly, Short-read, Long-read, Mapping, Hybrid, RNA-Seq, Gene fusion

## Abstract

Recent research into structural variants (SVs) has established their importance to medicine and molecular biology, elucidating their role in various diseases, regulation of gene expression, ethnic diversity, and large-scale chromosome evolution—giving rise to the differences within populations and among species. Nevertheless, characterizing SVs and determining the optimal approach for a given experimental design remains a computational and scientific challenge. Multiple approaches have emerged to target various SV classes, zygosities, and size ranges. Here, we review these approaches with respect to their ability to infer SVs across the full spectrum of large, complex variations and present computational methods for each approach.

## Introduction

Structural variants (SVs) are large genomic alterations, where large is typically (and somewhat arbitrarily) defined as encompassing at least 50 bp. These genomic variants are typically classified as deletions, duplications, insertions, inversions, and translocations describing different combinations of DNA gains, losses, or rearrangements [1–3]. Copy number variations (CNVs) are a particular subtype of SVs mainly represented by deletions and duplications (reviewed in Carvalho and Lupski [4]). SVs are typically described as single events, although more complex scenarios involving combinations of SV types exist [5, 6]. Chromothripsis, which is a large and complex combination of rearrangements reported in cancer [7], is an example. While the average genomic variation between two humans is 0.1% in terms of single nucleotide variants (SNVs), when taking SVs into account, this increases to 1.5% [8]. In particular, telomeric regions are affected by a higher rate of SVs [9].

SVs can have a pronounced phenotypic impact—disrupting gene function and regulation or modifying gene dosage. Multiple studies have highlighted their role in functional changes across populations [1, 10, 11] and species [12]. Their importance in medicine and molecular biology has been highlighted by multiple recent studies. For instance, in neurological diseases, SVs have been often discussed based on ATTCC repeat extensions in Parkinson [13] or CAG expansions in Huntington disease [14]. Furthermore, a retrotransposon insertion in an intron of the TAF1 gene has been associated with early stages of linked dystonia-parkinsonism disease [15]. In cancer, different types of SVs have been highlighted as causing various types of dysfunction: (i) deletions or rearrangements truncating genes [16]; (ii) amplification of genes leading to overexpression, for example, due to homologous recombination (HR) that leads to an inactivation of BRCA1 and BRCA2 [17, 18]; (iii) gene fusions, such as Her2-positive SKBR3 breast cancer that combines multiple genes across chromosomes [19]; and (iv) alteration of the location of gene regulatory elements, causing changes in the gene expression [4, 20]. In Mendelian studies, multiple diseases have been associated with deletions or duplications of genic regions. For example, three complex SVs affecting ARID1B (Coffin-Siris syndrome), HNRNPU (hypotonia), and CDKL5 (early infantile epileptic encephalopathy is a severe intellectual disability and Rett-like features) have been reported [21]. Another more recent study showed the complexity of these CNVs and an increase in mutation rates for Potocki-Lupski and Smith-Magenis syndrome [22].

SVs are also playing an essential role in plants including having a direct phenotypic impact [23]. For example, SVs play important roles in tolerance for multiple plants: (i) in maize, a tandem triplication over the AMTE1 genes is reported to be associated with aluminum resistance [24]; (ii) an amplification of Bot1 plays an important role in boron toxicity in barley [25]; and (iii) for weeds, a tolerance against the herbicide glyphosate based on amplification of EPSPS has been reported in response to extensive use of glyphosate [26]. Other SVs have a positive impact on fruit yield and quality. For example, a transposon insertion near Ruby, a MYB transcriptional activator, leads to the increase of anthocyanin concentration in blood orange compared to pumelo and mandarin [27]. In tomatoes, a transposon insertion in JOINTLESS2 (J2) results in undesirable branching of flower-bearing shoots (inflorescences) in genetic backgrounds that also carry a cryptic variant for the close homolog enhancer of J2. This combination results in excessive flower production. However, an additional tandem duplication in fresh-market breeding lines across this region leads to a threshold of correctly spliced product and thus to a healthy phenotype with higher fruit yield [28].

**Fig. 1:**
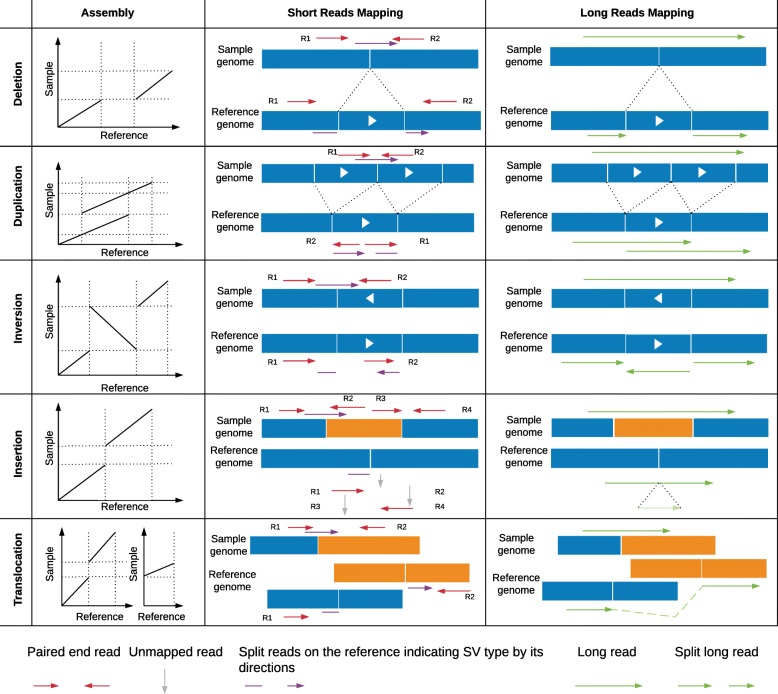
Comparison between de novo assembly, short-read and long-read mapping approaches to identify structural variants. For de novo assembly approaches, the relative positions of the segments in the dot plot indicate the type and size of the SV. For short-read-based mapping approaches, paired-end (red) and split reads (purple) are typically used to decipher the type size and location. In addition, the coverage can be used to improve the detection of deletions and duplications. Long-read-based mapping approaches typically leverage the alignment patterns of long reads (green) to detect the different types of SVs

Despite all these evidences of the importance of SVs, they have been largely understudied, compared to SNVs, because they are much more difficult to identify. In principle, taken individually, each type of SV induces a distinctive pattern in mapping reads that can be used to infer the underlying mutation. For example, a deletion forms a lack of a sequence and thus a gap in the alignment of the sample relative to a reference (Fig. 1). However, in practice, it is much more complicated. First, sequencing and mapping errors blur the patterns. Indeed, in contrast to SNVs and smaller insertions and deletions, SVs can cover a large portion of a read or even be larger than the read length—which complicates mapping [5]. Second, the patterns induced by the different SV types can be very similar. For example, it is often hard to distinguish tandem duplications from novel insertions for genomic alignments (Fig. 1). Finally, multiple SVs can overlap or be nested, giving rise to much more complex mapping patterns than when considered individually [5, 20]. Such complex patterns may preclude mapping altogether, forcing researchers to assemble each genomic sample de novo—a difficult and more costly task with conventional sequencing.

However, great strides have recently been made, thanks to technological and methodological developments. The advent of long-read sequencing technology, in particular, Pacific Biosciences (PacBio) and Oxford Nanopore technologies (ONT), makes it possible to produce reads of several thousand base pairs, even reaching up to 2 Mbp for Oxford Nanopore [29]. Furthermore, as we shall review in more detail below, technologies such as linked reads (e.g., 10x Genomics), optical mapping, and Strand-Seq have also been developed to improve the quality of assemblies and/or SV calling. Long reads help to increase the detection of SVs as they considerably ease de novo genome assembly and mapping. Nevertheless, the increased length and the higher error rate of emerging long-read technologies can pose new methodological challenges. Complementary to long reads, another noteworthy development has been the repurposing of transcriptomics (RNA-Seq) to detect SVs—in particular, rearrangements. Indeed, by identifying apparent RNA fusions, which are thus inherently transcribed, it is possible to focus on SVs with potential functional implications. Lastly, recent progress in benchmarking is greatly improving our understanding of the strengths and weaknesses of each approach. Current efforts such as Genome in the Bottle [30] and the FDA-led initiative SEQC2 (https://www.accessdata.fda.gov/scripts/fdatrack/view/track_project.cfm?program=nctr&id=NCTR-DBB-PM-SEQC2-Phase-II) aim at better characterizing false positives and false negatives in SV calling.

**Fig. 2:**
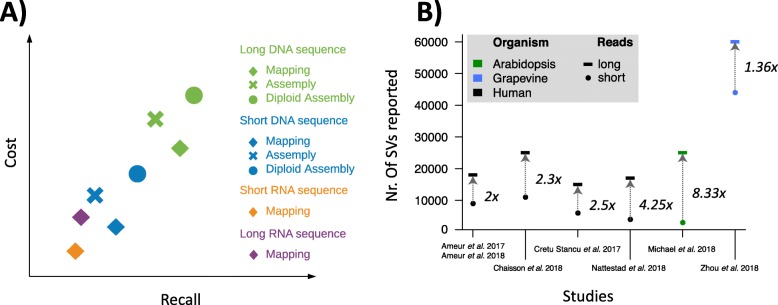
Qualitative overview of structural variant calling methodology using short reads and long reads and their associated costs. **a**, A qualitative comparison of the different SV methodologies ranging across technologies (whole genome and RNA-Seq using short and long reads) to different approaches (mapping vs. assembly) with respect to their costs and recall. **b**, The ratio of improvement in the number of SVs detected from using long reads across four human and two non-human studies. Overall, each study shows a clear improvement of using the longer reads. Additional file 1: Table S1 shows the details of each study

In this review, we give an overview of methods to detect SVs utilizing DNA and RNA-Seq from both short and long reads (Fig. 2). We provide a snapshot of the main methods currently available for detecting SVs (Table 1), with practical guidance as to which approach is suitable for which type of study. We conclude the review with a discussion of open challenges and future directions.

**Table 1:** Overview of multiple methods representative for the different SV methodologies currently used. Input types indicate the required data at start being either: De novo assembly (a), Oxford Nanopore (o), PacBio (p), 10X Genomics (x), Hi-C (h), Strand-Seq (t), Optical mapping (c) or Short reads (s)

Category	Name	Input types (a, c, h, o, p, s, tx)	Description	Link	Paper
De novo assembly	Cortex	s	Insertions, deletions, combinations of SNVs—inversions and deletions—rearrangements	http://cortexassembler.sourceforge.net/	[31]
	SGVar	s	Large insertions and deletions, complex SV		[32]
	HySA	p, s	Small (11 to 50 bp) to large (> 50 bp) insertions and deletions, complex SV	https://bitbucket.org/xianfan/hybridassemblysv/overview	[33]
	Assemblytics	a	Insertions and deletions (1 bp to 10 kb), repeat expansions/contractions	https://github.com/MariaNattestad/Assemblytics	[34]
	Paftools	a	Insertions, deletions	https://github.com/lh3/minimap2/tree/master/misc	[35]
	Smartie-sv	a	Insertions, deletions, inversions	https://github.com/zeeev/smartie-sv	[12]
	BreaKmer	s	Insertions, deletions, translocations, inversions, duplications	https://github.com/ccgd-profile/BreaKmer	[36]
	novoBreak	s	Deletions, duplications, inversions, translocations	https://sourceforge.net/projects/novobreak/	[37]
Short-read mapping	BreakDancer	s	Deletions, insertions, inversions, intra-chromosomal and inter-chromosomal translocations	https://github.com/genome/breakdancer	[38]
	BreakSeq		Insertions, deletions, translocations, inversions, duplications	http://sv.gersteinlab.org/breakseq/	[39]
	CREST	s	Insertions, deletions, translocations, inversions, duplications	https://www.stjuderesearch.org/site/lab/zhang	[40]
	DELLY	s	Deletions, inversions, duplications, inter-chromosomal translocations	https://github.com/dellytools/delly	[41]
	EricScript	s	Gene fusion	https://sourceforge.net/projects/ericscript/	[42]
	FusionCatcher	s	Gene fusion	https://github.com/ndaniel/fusioncatcher	[43]
	GRIDSS	s	Insertions, deletions, translocations, inversions, duplications	https://github.com/PapenfussLab/gridss	[44]
	Gustaf	s	Deletions, inversions, duplications, translocation	http://www.seqan.de/apps/gustaf/	[45]
	IDP-fusion	p, s	Gene fusion	https://www.healthcare.uiowa.edu/labs/au/IDP-fusion/	[46]
	JAFFA	p, s	Gene fusion	https://github.com/Oshlack/JAFFA/wiki	[47]
	LUMPY	s	Deletions, duplications, inversions, translocations	https://github.com/arq5x/lumpy-sv	[48]
	Manta	s	Insertions, deletions, translocations, inversions, duplications	https://github.com/Illumina/manta	[49]
	Meerkat	s	Insertions, deletions, translocations, inversions, duplications	http://compbio.med.harvard.edu/Meerkat/	[50]
	Pindel	s	Insertions, deletions, translocations, inversions, duplications	https://github.com/genome/pindel	[51]
	STAR-Fusion	s	Gene fusion	https://github.com/STAR-Fusion/STAR-Fusion/wiki	[52]
	SQUID	s	Gene fusion	https://github.com/Kingsford-Group/squid	[53]
	TARDIS	s	Discovery of tandem and interspersed segmental duplications	https://github.com/BilkentCompGen/tardis	[54]
	TIGRA	s	Insertions, deletions	https://bitbucket.org/xianfan/tigra	[55]
	Tophat-Fusion	s	Gene fusion	http://ccb.jhu.edu/software/tophat/fusion_index.shtml	[56]
	Ulysses	s	Insertions, deletions, translocations, inversions, duplications	https://github.com/gillet/ulysses	[57]
	SvABA	s	Insertion, deletions, somatic rearrangments	https://github.com/walaj/svaba	[58]
Long-read mapping	NanoSV	o	Local SV (LSV): duplications, deletions, inversions; insertions (transposons, intra-chromosomal (> 1 Mb away) and inter-chromosomal insertions)	https://github.com/mroosmalen/nanosv	[59]
	PBHoney	p	Insertions, deletions, duplications, inversions, translocations	https://sourceforge.net/projects/pb-jelly/	[60]
	PBSV	p	Insertions (20 bp to 5 kb), deletions (20 bp to 100 kb), inversions (200 bp to 5 kb), intra-chromosomal (> 100 kb away) and inter-chromosomal translocations, complex SV	https://github.com/PacificBiosciences/pbsv	
	SMRT-SV	p	Insertions, deletions, duplications, inversions, translocations	https://github.com/EichlerLab/pacbio_variant_caller	[61]
	Sniffles	o, p	Insertions, deletions, translocations, inversions, duplications, complex SV (nested SV)	https://github.com/fritzsedlazeck/Sniffles	[62]
Multimethods SV caller	FusorSV	s	Combining LUMPY, DELLY, and GenomeSTRiP	https://github.com/TheJacksonLaboratory/SVE	[63]
	MetaSV	s	Combining BreakSeq, Breakdancer, Pindel, CNVnator	http://bioinform.github.io/metasv/	[64]
	Parliament2	s	Combining LUMPY, DELLY, Manta, BreakSeq, CNVnator	https://github.com/dnanexus/parliament2	[65]
	SURVIVOR	a, o, p, s	Can combine/compare any SVs VCF	https://github.com/fritzsedlazeck/SURVIVOR	[10]
Hi-C technology	Hic_breakfinder	h	Detects SVs based on optical mapping, Hi-C, short reads	https://github.com/dixonlab/hic_breakfinder	[66]
	HiCnv	h	Pipeline to identify CNVs from Hi-C data	https://github.com/ay-lab/HiCnv	[67]
	HiCtrans	h	Identify potential translocations using change-point statistics	https://github.com/ay-lab/HiCtrans	[67]
Optical mapping		c	Commercial tools; visualization and analysis of Bionano data	https://bionanogenomics.com/support-page/bionano-access-software/	
Strand-Seq technology	Strandseq-InvertR	t	R package to locate putative inversions	https://sourceforge.net/projects/strandseq-invertr/	[68]
10x Genomics	Gemtools	x	Downstream and in-depth analysis of SVs from linked-read data	https://github.com/sgreer77/gemtools	[69]
	GROC-SVs	x	Identify large-scale SVs based on barcode information	https://github.com/grocsvs/grocsvs	[70]
	LongRanger	x	Align reads, call and phase SNPs, indels, identify SVs	https://support.10xgenomics.com/genome-exome/software/downloads/latest	[16]
	NAIBR	x	Identifies novel adjacencies created by SVs events	https://github.com/raphael-group/NAIBR	[71]

**Table 2:** Glossary**.** Here, positive (P) or negative (N) describes the SV detection (or SV calling), and true (T) or false (F) describes if the calling was correct. Thus, SVs are true positive (TP) if they are called or false negatives (FN) if they are not called but present in the sample. Conversely, SVs that are not in the sample are true negatives (TN) if they are not called or false positives (FP) if they are called

Word	Definition
Accuracy	Proportion of correctly identified events (T) to the overall events: (TP + TN)/(TP + TN + FP + FN).
Breakpoints	Positions on the genome denoting the start and end of SVs relative to the reference genome.
Contigs	Contiguous sequence stretches assembled from reads.
De Bruijn graph	Directed graph consisting of nodes with exactly *n* incoming and *n* outgoing edges. In genome assemblies, a de Bruijn graph is built where the nodes are *k*-mers (sequences of length *k*) and the edges correspond to the overlap on *k* − 1 bases between nodes.
String graph-based assembly	Similar method to De Bruijn graph-based assembly, but in this case, the overlaps between all read pairs (instead of *k*-mers) are computed to construct a string graph based on the overlaps.
Insert size	The distance between the two paired-end reads.
Overhang	Portion of a mapped read that cannot be aligned and thus could indicate a structural variation.
Phasing	The identification of two or more heterozygous variations are co-occurring on the same or different DNA molecule.
Precision (or positive predictive value)	Proportion of predictions (FP + TP) that are correct (TP).
Recall (or sensitivity or true-positive rate)	Proportion of the total positives (FN + TP) that were correctly identified (TP).
Scaffold	Connected contiguous sequence stretches, with unresolved sequence stretches in between.
Split reads	Reads containing parts that map in different loci on the reference genome. They are found by splitting the read in sub-segments, align individually each sub-segment, and then grouping sub-fragments from one read.
Tandem sequence	A specific type of repetitive region that was repeated directly adjacent to each other.

## De novo assembly-based approach

De novo genome assembly has traditionally been used to generate reference genomes. Multiple strategies have been proposed, utilizing long and short reads or leveraging both. We refer the interested reader to the review of Nagarajan and Pop [72], which provides a critical overview of de novo assembly methods.

To detect SVs, such de novo-assembled sequences can be aligned to a reference or other assembly (Fig. 1), and the alterations between the two can be systematically identified: the comparison of each position in one genome to its corresponding position in the other genome should allow the identification of all forms of variations [3, 73]. Discontinuities that arise from certain types of SVs during a whole-genome alignment result in different patterns (Fig. 1). However, although conceptually simple, genome alignment is computationally far from trivial [74].

Multiple methods have been proposed to identify SVs based on a genomic alignment. These can be distinguished by whether they construct an assembly graph or operate directly on the already assembled sequences. Methods that construct the assembly graph are typically slower, but can provide more insights, as they are leveraging the read information directly. Cortex is one of these methods that use short-read sequencing data and can simultaneously assemble several genomes. Cortex uses a colored de Bruijn graph (see Table 2 for definition) to simultaneously infer SVs and complex combinations of SNVs, indels, and rearrangements [31]. SGVar [32] is a more recent string graph-based (see Table 2 for definition) de novo assembly pipeline based on the SGA assembler [75] that also uses short-read sequencing data. SGVar uses a stringent read preprocessing based on the read length and read quality. It requires a perfect match to merge reads or sequences, which improves the assembly quality. Using both simulated and real data (chromosome six of the human genome), SGVar has been shown to outperform other methods, such as Cortex, for insertion and deletion identification [32].

The other group of methods operate based on previously assembled contigs or scaffolds and are thus independent of the sequencing technology (see Table 2 for definition). Basically, they rely on alignments between an assembly and a reference assembly, computed with aligners such as BlasR [76], MUMmmer [77], or Minimap2 [35]. Assemblytics [34] is a web application that relies on MUMmer and identifies insertions and deletions up to 10 kbp. It distinguishes between contractions and expansions of repetitive elements in contrast to insertions and deletions in a unique sequence. This can be an important distinction since it already annotates the type of event to provide further insight. Another method paftools.js [35] uses Minimap2 alignments, which are typically many fold faster than MUMmmer-based approaches. Similar to Assemblytics, it calls insertions and deletions but only runs on the command line. SMARTie-SV was recently introduced to detect insertions, deletions, and inversions, using BlasR. It has been applied to study SVs across great apes (gorillas, chimpanzees, orangutans) and humans [12].

Theoretically, all forms of structural variants can be identified given a fully contiguous and complete de novo assembly. The main strength of de novo assembly-based approaches compared to other approaches lies in detecting larger insertions (3+ kbp) [34, 32]. One major challenge is the lack of haplotype representation. Thus, heterozygous SVs are often missed simply by the fact that a standard de novo assembly only represents one haplotype. Nevertheless, there are de novo assembly methods to account for this such as trio-sga [78], Falcon-Unzip [79], or Trio-Canu [80] that often require additional coverage and/or parental information. They can provide diploid information of the genome and thus enable a better representation of heterozygous SVs. However, some challenges remain even for a haplotype representation, such as the de novo assembly quality and improving the genomic alignments by taking a larger genomic context sequence into account. Therefore, the de novo assembly-based approach is often used for a small number of challenging samples or for studying organisms that do not have a genome of reference.

## Short-read alignment approach

Short paired-end sequencing data dominates most of the publicly available datasets. Typically, these paired-end reads are mapping in the opposite orientation and within a certain distance of each other (e.g., 500 bp). In the presence of SVs, these pairs are abnormally oriented and or spaced (Fig. 1). In addition, split reads can be used to improve the breakpoint resolution (Fig. 1). SV calling using paired-end reads is currently the standard approach and has been applied to single samples up to large cohorts (e.g., 1000 genomes).

In this section, we first focus on DNA-Seq-based methods then on RNA-Seq-based ones.

### Short-read DNA-Seq mapping

Over the past decade, more than 100 short read-based mappers have been introduced, yet read mapping is still not entirely solved—for example, when it comes to reliably aligning reads to highly polymorphic regions [81]. Once the reads are mapped, their insertion size, orientation, and alignment length can be used to identify SVs candidates. Figure 1 gives a detailed overview of the patterns of abnormally mapped paired reads and how they relate to SVs types. For example, a deletion in a sequenced sample leads to a larger insert size (the distance of the pairs). In addition, the coverage in the allele is half (heterozygous) or zero (homozygous) compared to the surrounding regions. For duplications, the coverage is increased, and for rearrangements, the pairs are abnormally spaced or oriented while the coverage is not affected. This signal is often filtered by coverage, mappability, or other measurements, such as an increase in substitutions.

The methods for detecting SVs from short reads vary in the type of information they exploit. Early methods relied exclusively on the distance and orientation of paired-end reads (Fig. 1). For example, BreakDancer [38] classifies each read into normal or SV depending on the mapping distance and orientation between the read and its mate. Regions with an excess of reads fitting into an SV category are then identified and assigned a confidence score. This can lead to missed variations, e.g., smaller deletions, for which the length is within the variability of the paired-end distribution. To increase the resolution, split reads can also be used. DELLY [41] integrates the analysis of split reads into its search of abnormal distances and orientations among pairs of reads. Although this increases the accuracy of breakpoint prediction and enables the detection of smaller deletions (20+ bp), the larger events remain hard to distinguish from mapping artifacts. To overcome this, some methods have integrated coverage information as a third kind of input signal. For example, LUMPY [48] does a joint analysis of the read depth, paired-end read discordance, and split-reads. Another tool that leverages all three types of information is Manta [49], which includes a highly parallel strategy that can be used on an individual sample or on a small set of samples including tumor-normal pairs. This is achieved by parallelly building graphs across regions of the genome and testing for a specific variant hypothesis. The nodes of such graphs are regions that may contain one or more breakpoints, and the edges represent the evidence (i.e., reads) of breakpoints between the regions (see Table 2 for definition). The evidence accumulated around every pair of genomic regions is then evaluated for specific SVs hypotheses. GRIDSS [44], on the other hand, retains only the reads that provide evidence for SVs and then assembles them via a positional *de Bruijn* graph. The alignment of the subset of reads enhances the accurate identification of SVs, thus achieving an increased recall. Regarding precision (the proportion of inferred SVs that are correct), GRIDSS’s authors show similar performance to LUMPY, with an estimated precision rate of 90% (evaluated from 1000 previously validated deletions) [44]. In the same study, BreakDancer, Pindel, DELLY, and Manta exhibited lower precision rates, ranging from 70 to 85%. However, GRIDSS has the disadvantage of reporting any type of SV event as a simple breakpoint (i.e., BND), and this makes the interpretation of the underlying SV type difficult. More recently, to detect more complex events such as a tandem duplication where the second copy is inverted, methods such as TARDIS have been proposed [54].

The aforementioned methods specialize in the detection of specific types of variants, but none of them is able to reliably identify all SV types and size regimes [5, 10, 82, 83]. Meta-methods seek to fill in this gap by combining calls from different tools and selecting the variants identified by multiple methods. Ideally, meta-methods can combine the strengths of multiple methods while overcoming their individual weaknesses. In practice, this works up to a certain point, but these methods can also serve to adjust the precision-recall trade-off more flexibly. MetaSV [64], Parliament2 [65], and SURVIVOR [10] have been reported to yield higher recall than a single caller, at the cost of moderately reduced precision. Using different parameters, SURVIVOR can also be used to increase precision, at the cost of a moderately reduced recall [10, 19]. Furthermore, SURVIVOR can also incorporate the information from short and long reads to further improve precision and recall.

Overall, short-read-based methods are well established and widely used. Nevertheless, the recall is often reported to be between 10 [61] and 70% [1, 5, 10] and the false-positive rates are very high (up to 89%) [60, 73, 84, 85] depending on the size and type of SVs. While rearrangements or certain larger (500+ bp) deletions are robustly identified, mid to larger size insertions remain a major challenge. These insertions are often disturbing the accurate alignment of reads and thus can lead to misinterpretations [5]. These cases might be resolved by using a localized assembly approach, for example using SvABA [58]. In addition, these methods are often blind to certain regions (e.g., low complexity, highly repetitive, highly mutated) of the genome. To sum up, while we can control the precision of these short-read-based methods, the recall can only reach a certain point and certain complex types of SVs will remain hidden [1, 5, 19, 82]. Thus, we may be reaching the limits of DNA mapping approaches based on short reads. Indeed, the emergence of meta-methods may well be indicative of diminishing returns in a maturing field.

### RNA-Seq mapping

In contrast to the genome approaches, RNA-Seq-based approaches focus only on expressed regions. Here, the challenges are different, and thus, specialized methods have been proposed. In general, RNA-Seq methods aim to identify gene fusions, which are connections between parts or full lengths of two or more genes. Using RNA-Seq, we can detect if the variant observed is expressed and measure the amount of expression in comparison with other genes.

Multiple methods have been developed to detect gene fusions. These methods work based on mapping of short RNA-Seq paired-end reads to the reference genome and or transcriptome. Subsequently, the abnormal spaced paired and split reads (see Table 2 for definition) between different genes are identified, summarized, and filtered. Recent benchmarks highlighted the impact of the read quality and length to detect gene fusions but disagreed about their recommendation [46, 86–88].

For gene fusion detection, the methods mainly differ in how strictly they use existing gene annotations. Reliance on gene and exon annotations can increase precision by disregarding or correcting mapping errors. For instance, methods such as FusionCatcher [43] and EricScript [42] inherently focus on the annotated parts of the genome. FusionCatcher is designed to identify somatic fusion genes, by aligning reads to a transcriptome using Bowtie [89] guided by Ensembl annotation. It removes the reads that align to rRNA and tRNA or trim them if they have a low base quality to improve the prediction of gene fusions. EricScript follows a novel approach mapping first the paired-end reads and performing a localized assembly across fusion candidates to obtain better exon junction candidates. The reads are then mapped back to the fusion catalog, and annotation candidates are subsequently scored and filtered.

On the other hand, methods that do not strictly rely on the annotation of a genome can have a higher sensitivity. Indeed, annotations are typically incomplete, even for well-characterized organisms such as humans [90], let alone for non-model organisms. A loose reliance on annotations is further relevant when dealing with cancer samples [19], which can contain complex non-canonical gene fusion patterns. One of the earliest fusion detection methods was TopHat-Fusion [56], which used a specialized version of TopHat [91]. Of note, TopHat is outdated, and its authors recommend to use HISAT2 [92] instead. STAR-Fusion [52] is leveraging the speed and accuracy of the STAR RNA-Seq aligner [93] by selecting parameters optimized for gene fusion detection (e.g., allowing chimeric alignments, setting a low minimum overhang for a chimeric junction) (see Table 2 for definition). STAR-Fusion uses single or paired-end reads mapped to a reference and annotation index. SQUID [53] constructs a graph based on the regions with discordant reads. The graph represents candidates of gene fusions and the reference where the individual neighboring regions (nodes) are connected. The connections are subsequently weighted by the number of supportive reads. Linear programming is then used to traverse the graph and report gene fusions.

The last group of RNA-Seq fusion detection methods has been conceived to also take advantage of long reads—in particular, those obtained from the PacBio Isoform Sequence protocol. IDP-fusion [46] and Jaffa [47] are gene fusion identification tools that consolidate long-read with short-read RNA sequencing data. IDP-fusion requires both long and short reads while it is optional for Jaffa. The long reads are used primarily to identify fusion candidates. Subsequently, short reads are used to improve the breakpoint accuracy and precision.

Overall, RNA-Seq-based SV detection has the advantage of determining if an allele is expressed or not. Although this is no guarantee that this variant has an impact on the phenotype (the protein might not be translated or stable), RNA-Seq helps with prioritizing fusions that affect gene structure. However, there are multiple disadvantages. First, the underlying SV type can be uncertain for the gene fusion. This might complicate the interpretation, as well as the validation. Second, the coverage levels vary with the expression of the gene. Thus, lower expressed genes and their variations are likely to be missed. Third, SVs that impact promoter regions, introns, or non-transcribed regions are not detectable. This is especially the case for some of the methods penalizing read mapping outside of annotated regions. And fourth, previous benchmarks have shown that gene fusion studies often suffer from high false-positive rates, for example, due to chimeric regions [94].

## Long-read mapping-based approach

Long reads are advantageous for SV calling because they can span repetitive or other problematic regions. Thus, these longer reads (5+ kbp) have the potential to improve the mapping and also to capture larger SVs better compared to short reads alone [5, 60, 76, 82, 83]. Both PacBio and Oxford Nanopore methods can generate reads of thousands of base pairs but present two major disadvantages. First, the costs for sequencing are higher to obtain the same coverage compared to short-read sequencing. Second, the high sequencing error rate (~ 8–20%) [95] has to be considered for both alignment and SV calling steps. Thus, specialized methods to align long reads such as BLASR [76], Minimap2 [35], and NGMLR [5] were recently developed. The identification of SVs is still at an early stage with only a few methods available.

With long reads, the SV detection methods are often tailored to the underlying technology—mainly PacBio or Oxford Nanopore. One exception is Sniffles [5], which employs a parameter estimation in the beginning and thus adjusts itself to the underlying error model. Sniffles operates on a per read base, also capable of reporting very low-frequency SVs in the sample. This is particularly useful in cancer or in mosaic variation. Furthermore, Sniffles allows the detection of more complex or adjacent SVs such as inversions flanked by deletions or inverted tandem duplications. It implements a statistical framework to reduce the number of false-positive calls.

For PacBio, three main specialized methods have been proposed. PBHoney [60] uses coverage and split read information relying on BLASR alignments. PacBio structural variant calling and analysis tools (PBSV) is a method developed by PacBio to detect SVs within the range of 20+ bp (https://github.com/PacificBiosciences/pbsv). Reads supporting a putative SV are used to generate a consensus, which is then re-aligned to the reference genome. SMRT-SV [61] includes de novo assembly and a specialized genotyping module. Reads are first aligned to the reference and, subsequently, a local assembly is performed for each multiple kbp window across the entire genome. The resulting assemblies are then aligned back to the reference, and structural variants (insertion, deletions, and inversions) are identified.

For Oxford Nanopore, NanoSV was one of the first methods developed [59]. NanoSV preferentially uses as input an alignment from LAST [96], which uses adaptive seed rather than fixed-length seed for speed optimization [96]. Of note, NanoSV reports only breakpoints (BND) which again makes the interpretation of the SVs type difficult.

Overall, long-read mapping-based methods for SV calling often show a better performance than short-read ones (Fig. 2). Indeed, longer continuous reads can be aligned more accurately, even after accounting for the higher sequencing error rate. Furthermore, the enhanced length enables a full capture of most of the alleles for SVs—in contrast to short reads where multiple pieces of information have to be put together to infer single SVs. However, there are still some performance deficiencies for larger (5+ kbp) insertions compared to de novo assemblies. This is because, as with short reads, the allele is getting longer than the read itself. Current efforts perform a localized assembly to improve, but do not fully solve, this issue when looking at very large insertions or inversions that are flanked with large low-complexity repeats (e.g., 5 kbp). Nevertheless, multiple papers have reported a significant improvement in precision and recall for SV calling using long reads compared to short-read mapping approaches [2, 5, 19, 82, 97–99].

## Alternative approaches for the identification of structural variants

While this review focuses on SV calling methods utilizing short and long reads, there are other technologies that have recently improved our ability to call SVs. In this section, we provide a brief overview of these technologies and the associated software packages and refer the interested reader to other reviews for more details [95, 100–102].

Linked reads produced by 10x Genomics enable to pair reads over distances of up to 150 kb, and multiple methods have been developed to detect SVs from the linked reads. The challenge here is to identify an SV based on sparse coverage of the molecule with paired-end Illumina reads. These methods typically have a specific target SV size resolution because the barcode identifying the paired-end reads per molecule is not unique and the distance between the individual paired-end reads is undefined. Prominent methods for this technology include LongRanger [16] (50+ bp for deletions, 30+ kbp for rearrangements), GROC-SVs [70] (min 10 kbp) utilizing a localized assembly, and NAIBR (1+ kbp) [71], which uses a probabilistic model that combines multiple signals in barcoded reads.

Another technology relying on short-read sequencing is Hi-C, which is used to identify regions that are in close proximity in 3D space, which provides longer-range information than standard short read. An alteration of these pairs is likely caused by an SV allele at the location. Several methods have been devised to directly detect SVs based on Hi-C data. While some methods, such as Hic_breakfinder (1+ Mbp), can potentially identify all types of SVs [66], others, such as HiCnv (> 1 Mbp) and HiCtrans [67], only aim to detect CNVs and translocations, respectively.

Strand-Seq is a new sequencing method that preserves strand directionalities. Thus, when the reads are aligned to the reference genome, the individual homologs for each chromosome can be distinguished [101]. This helps in identifying inversions, for example, using Strandseq-InvertR [68] (min ~ 1 kbp), and can also be applied at a single-cell level.

Optical mapping, e.g., provided by BioNano, uses a different approach based on restriction enzyme maps which labels 7-bp markers. Optical mapping is a highly cost-efficient method to detect SVs but is often limited in terms of breakpoint accuracy and in terms of distinguishing SVs that are close to one another. Furthermore, BioNano cannot provide the sequence of an allele (e.g., insertions). SV calling from BioNano data can be performed using the vendor’s software, called BioNano Access (https://bionanogenomics.com/support-page/bionano-access/).

## Discussion

SVs are increasingly being recognized as an important class of variants, which need to be considered in evolutionary, population, and clinical genomics. In this review, we delved into different available algorithms to call SVs, highlighting their advantages and disadvantages. It transpires that SV calling methods based on short-read mapping offer a cost-efficient way to search for most known SV alleles (genotyping) [103], but they struggle to detect novel SVs, especially insertions [5, 82, 83]. On the other hand, SV calling approaches from de novo assembly require a contiguous, haplotype-resolved and complete representation of the sample, something which can only be achieved through costly high-coverage sequencing. This makes them currently impractical when dealing with multiple samples (e.g., > 20)—which, for instance, is needed for population-scale studies. However, they are necessary to reliably detect and resolve complex SVs alleles. As for the long-read-based SV mapping approaches, they are at the “bleeding edge”. Long-read sequencing is currently more expensive and less widespread than short-read sequencing. However, this is currently changing with continuous reductions from both Oxford Nanopore and PacBio cost per base. It is already apparent that SV calling from long-read mapping can be more effective than from short-read mapping approaches. In addition, mapping approaches are often less expensive than de novo assemblies. For applications requiring the elucidation of very long or very complex SVs, it is still possible to perform a localized long-read de novo assembly. Phasing SVs can further improve the overall quality by identifying which SVs violate the diploid genome assumption. Clearly, this needs to be adopted, given copy number alterations or genomes with higher ploidy. Due to the complexity, only few studies were able to do this so far with a success of 78.7%, even though parental genomic data was used [59].

Regardless of the sequencing technology and SV calling algorithm, a challenge that remains is the comparison and interpretation of SVs. For example, a tandem duplication will result in having the second paired read or part of the read mapped before the first (Fig. 1). Interspersed duplications induce very different mapped read patterns, which can easily be confounded with an inversion or deletion (if the duplication is on the same chromosome) or with a translocation (duplication on a different chromosome). This is caused by molecules that have recombined between different regions, an event which can occur in cancer. In such cases, the reads of these regions will map back to their original locations along the genomes, forming larger gaps in their alignments. These gaps are then misinterpreted sometimes as different SV types flanking the duplicated regions, depending on their distance to each other (Fig. 1). As for insertions, while a novel sequence will indeed be identified as an insertion, a sequence that is similar to a region in the genome (e.g., 80% identity or more) can be called depending on the location of the region as a translocation, inversion, or deletion event. Lastly, when comparing de novo assembly-based calls and mapping-based calls, duplications and insertions can be hard to distinguish: while a genomic alignment may indicate a novel sequence between two genomes, mapping-based approaches might highlight the same event as a tandem duplication if the inserted sequence shares similarity to the neighboring region. As these examples illustrate, comparing different SV call sets and reconciling them can add a whole new layer of difficulty to the problem.

For methods to progress, benchmarking is critical. Currently, the performance of each method remains hard to assess, because precision and recall are typically estimated on different datasets, each presenting different challenges, often using inconsistent operational definitions (e.g., a minimum length of 20 vs. 50 bp to be considered a SV). Furthermore, most benchmarks to date are limited to simulated datasets: this is advantageous in that the truth is known with certainty, but it is often unclear how such results generalize to real datasets. To establish gold standards and facilitate the comparison of different methods, several efforts are underway, such as Genome in a Bottle (led by the US National Institute of Standards and Technology) and SEQC2 (lead by the US Food and Drug Administration). Both seek to obtain a better gold standard and understanding of the underlying bias. This is achieved by sequencing trios very deeply with multiple technologies (Genome in a Bottle) or sequencing a sample multiple times by different laboratories and different sequencing machines (SEQC2). The results of these studies will further highlight the advantages of certain approaches over others.

Ultimately, for SVs to be routinely considered in evolutionary and medical studies, standard methods and reference databases will be required. An improved differentiation between germline and somatic SVs would be desirable, similar to that of SNVs, to improve the categorization of SVs. Currently, only few methods exist that offer an initial assessment (e.g., Manta [49]). Databases of allele frequencies such as gnomAD [104] are available for SNVs, but we completely lack them for SVs. The annotation of SVs is often more difficult because their length needs to be taken into account, and the underlying sequence itself needs to have a reliable allele frequency assessment. Furthermore, although SVs can be reported using the standard Variant Call Format (VCF), there are inconsistencies in the way different methods report SVs. Some methods fail to report sufficient information to determine the exact type of SV or report valuable extra information in an ad hoc format. Standardization would greatly facilitate SV calling across multiple samples. One possible solution would be to extend the format in a similar way as with the Genomic VCF format (gVCF) for SNVs. In that format, for SNVs and smaller insertion and deletions, the reference information is also included to enable subsequent genotyping of variants that might not have been called in the initial assessment. Such an approach greatly speeds up the assessment and often increases the accuracy.

Likewise, before SV calling becomes routine in clinical settings, several challenges will need to be overcome. Besides the challenges in detection and correct genotyping, we are lacking an assessment and annotation of SVs. One of the best indicators if a variant is a candidate for pathogenicity is if this variant occurs at a low frequency (e.g. < 0.5%) in the population. While it is possible to assess the frequency of SNVs using reference datasets such as gnomAD/ExAC [104], this is much more difficult for SVs [103]. Indeed, while there is only a small number of possible SNVs at each site (typically one or two alleles, but only up to four given the nature of DNA), the number of possible SVs affecting each site is much larger, due to their size and type differences. This also complicates our ability to compare SVs with each other. Finally, because of the need for certification and quality assurance in a clinical setting, the aforementioned lack of format standardization and metadata information is even more acute in clinical applications than in research.

In conclusion, the current state of SV calling is akin to that of SNV calling about 10 years ago: its value is unquestionable, but the technology and methods are still evolving very rapidly, and the lack of standard protocols, benchmarks, and reference databases means that SV calls require careful interpretation. Considering the intense competition among long-read sequencing providers and the need for SV characterization for clinical applications—in particular for cancer diagnostic and treatment—it will not be long before SV analysis becomes routine.

## Supplementary information

**Additional file 1: Table S1.**

**Additional file 2:** Review history.
