# Supplementary material for: Structural variant calling: the long and the short of it
Source: Genome Biol. 2019 Nov 20;20:246. doi: 10.1186/s13059-019-1828-7 (PMC6868818; doi:10.1186/s13059-019-1828-7)
Supplement: Supplementary file 2 — Additional file 2: Review history. [file 13059_2019_1828_MOESM2_ESM.docx]

Review History

**First round of review**

**Reviewer 1**

**Comments to author:**

Structural variation are one of the most important types of genetic variation. They have been understudied and the approaches for discovery and genotyping them are still not as accurate as needed. The computational tools are very much dependent on the input data type and have different performance. This is a timely review and topic to work on. However, there are few review papers on SVs already published. It is not clear what aspect of SV prediction this review is focugin on. It would benefit if the message of the review was more clear. If it is about differences between results of short reads vs long reads for SV predictions then the results should be covered more. several major comments are as follow:

1. The review does not provide an overview of the computational signals and signatures used for predicting SVs. For example for short reads it is possible to use Read-Pair, Split-Read, etc.

2. There are several main computational formulations for predicting SVs. For example maximum likelihood, etc. These need to be covered in a comprehensive review.

3. There are major difference in SV predictions performance for short vs. long reads. Especially for different types of SVs (deletion vs duplication vs mobile element insertions vs more complex SVs).

4. Some new data types for SVs predictions are being used. These include Hi-C and 10X genomics. The review needs to cover these too. Especially what is the advantage of each of these datatypes for predicting SVs.

5. There are novel methods for predicting complex SVs (e.g., Inversion-Duplication) using short reads. They need to be covered.

6. A new study on SV insertion using 15 PacBio dataset was recently published (Audano et al. Cell). The results on this study should be covered. It gives a nice overview of the improvements using long-reads.

7. The potential application of phasing for better assembly and help in SV prediction should also be covered.

8. Recently mapping free approaches are being suggested for SV prediction They authors need to mention that too.

**Reviewer 2**

**Comments to author:**

This manuscript reviews different algorithms and approaches to SV calling (e.g. short vs. long reads, DNA vs. RNA seq) and the benefits and limitations of each. It describes the general difficulties in detecting SVs that are shared by all callers including inferring the SV type from the alignment, standardizing the output of SV callers, and genotyping SVs across populations. This is an important subject and the manuscript provides a good introduction to the benefits and challenges of SV calling and a useful table that lists each caller with the type(s) of SV it best detects and a link to download the code. However, the presentation could be clearer and more complete.

Major Comments

1. Structural variants are difficult to understand. Since this is a review, I would add some paragraphs towards the beginning describing different types of SVs and issues in calling them (e.g. determining location, amplitude of copy-number change, phasing). This would deserve a figure I think. Currently, the description is that SVs are “genomic differences”—unacceptably vague. Also, their description as >50 bp is entirely arbitrary and not a consistent convention. It would be reasonable to describe the range of alterations, and why they are split between indels, large SVs, and the SVs in between (largely because different algorithms have been used to detect indels vs large SVs, and the others have been a blind spot).

2. Indeed, I don’t think “phasing” is ever described in this review, despite its central importance in understanding the consequences of an SV. The ability to phase SVs would be a major selling point for any SV caller.

3. It is important to distinguish the specific challenges in calling somatic vs germline events and vice versa. E.g. purity and the “somatic” call in the case of somatic alterations; what to consider as the reference genome in the case of germline alterations. Alternatively, explicitly state (including in the title) that this review is focused on germline events.

4. The manuscript is focused on rearrangements and does not really mention their relation to copy-number alterations (also considered SVs), or the challenges and approaches to detecting telomeric alterations. For the latter, the manuscript should at least note that these exist and are the subject of a different set of algorithms, which will not be discussed here.

5. The focus on single events in the first paragraph should be expanded on: complex events have not only been reported on; they are common, especially (but not exclusively) in the somatic realm. Often they result from a single “event” that generates multiple rearrangements; this can be clarified. The manuscript should cite a chromothripsis paper here as well.

6. The importance of SVs should be highlighted more early in the introduction, rather than burying it in the middle of a paragraph. After all, they account for 15X as much variation between human genomes as SNVs (and something along these lines should be explicitly stated).

7. Box 1 doesn’t really describe the roles of SVs in medicine and biology (it really just focuses on the challenges in calling SVs). A box that really did this would be useful.

8. “De novo” assembly needs to be explicitly distinguished from local assembly approaches. The former include algorithms like DISCOVAR (which might be included in the manuscript); the latter include algorithms like MANTA (which is incorrectly classified as a mapping algorithm), novobreak (which is incorrectly classified as de novo assembly) and Svaba (which might also be included in the manuscript). Indeed, I would add local assembly algorithms as a separate section to the existing sections on de novo and mapping-based algorithms. It mediates between the two.

9. In the paragraph indicating that SVs have been understudied, perhaps the main reason has been left out: they largely require whole genome sequencing (and why this is more the case for SVs than SNVs can be explained). Also, that beyond difficulties in calling them, they can be difficult to understand because each SV can cover a large region of the genome and have long-range effects.

10. The description of long-range sequencing technologies should include 10X sequencing. This is becoming widely used and is conceptually different from PacBio/Nanopore.

11. In describing individual algorithms, it would be useful to indicate whether they work on somatic genomes, germline genomes, or both. This might be a column for the algorithms table, for instance.

12. BRASS and dRanger/Breakpointer might be included as additional short-read alignment algorithms. It would also be useful to refer to methods that integrate other datasets, such as Dixon et al Nat Gen 2018, who integrated HiC and optical mapping data to detect SVs.

13. I might include less description of individual methods in the text; the details could be left to tables or supplementary tables describing the different methods. The text should instead be used to make the overarching themes clearer, including the issues I described above.

Minor Points:

1. In the long reads mapping section, the following run-on sentence should be subdivided. “Sniffles operates on a per read base capable of reporting of reporting also very low frequency SVs in the sample, which is useful in cancer or for mosaic variation.”

2. The axes of the plot in Figure 2a do not have a scale

3. The caption in Figure 2b uses both word and numeric representations of numbers. It should consistently signify numbers through words (e.g. two) or numerals (e.g. 2).

4. There should be a heading for the Table after Figure 2

**Authors Response**

**Point-by-point responses to the reviewers’ comments:**

Reviewer #1:

Structural variation are one of the most important types of genetic variation. They have been

understudied and the approaches for discovery and genotyping them are still not as accurate as

needed. The computational tools are very much dependent on the input data type and have

different performance. This is a timely review and topic to work on. However, there are few

review papers on SVs already published. It is not clear what aspect of SV prediction this review

is focusing on. It would benefit if the message of the review was more clear. If it is about

differences between results of short reads vs long reads for SV predictions then the results

should be covered more. several major comments are as follow:

*We thank the reviewer for these comments. Indeed the review focuses on the up- and*

*downsides of assembly vs. mapping approaches for both long and short-read technologies.*

*These four different categories represent experimental designs with different trades-off in terms*

*of sample quality, sample numbers, availability of reference genomes, and costs.*

1. The review does not provide an overview of the computational signals and signatures used

for predicting SVs. For example for short reads it is possible to use Read-Pair, Split-Read, etc.

*The computational signal and signatures used for predicting SVs is provided in Figure 1, in*

*paragraph 3 in the introduction, and in the short read mapping sections.*

2. There are several main computational formulations for predicting SVs. For example maximum

likelihood, etc. These need to be covered in a comprehensive review.

*We thank the reviewer for this suggestion. While we have tried to provide methodological*

*insights where appropriate, our review is targeted to a broad audience of experimental and*

*Personal Cover Click here to access/download;Personal Cover;CoverLetter.docx*

*computational biologists. We have thus prioritized conceptual insights and practical guidance*

*over algorithmic considerations.*

3. There are major difference in SV predictions performance for short vs. long reads. Especially

for different types of SVs (deletion vs duplication vs mobile element insertions vs more complex

SVs).

*We agree with the reviewer. This is pointed out multiple times across the main text including*

*also Figure 1 and 2, and the Discussion section.*

4. Some new data types for SVs predictions are being used. These include Hi-C and 10X

genomics. The review needs to cover these too. Especially what is the advantage of each of

these datatypes for predicting SVs.

*We thank the reviewer for this excellent suggestion. We have added an entire new Box (Box 3)*

*to discuss these novel technologies (10x genomics, Hi-C, optical mapping and strand seq).*

5. There are novel methods for predicting complex SVs (e.g., Inversion-Duplication) using short

reads. They need to be covered.

*We agree and have incorporated TARDIS and other methods into the main text and Table 1.*

6. A new study on SV insertion using 15 PacBio dataset was recently published (Audano et al.

Cell). The results on this study should be covered. It gives a nice overview of the improvements

using long-reads.

*We agree and indeed we refer to this study multiple times throughout our review.*

7. The potential application of phasing for better assembly and help in SV prediction should also

be covered.

*We agree. Phasing is an emerging area, with currently very few methods that are able to phase*

*SVs directly. One method, which works for small to midsize deletion is WhatsHap. Another is*

*Crosstich, but it is still under development and is, as of yet, unpublished. We have extended the*

*discussion section to cover this.*

8. Recently mapping free approaches are being suggested for SV prediction They authors need

to mention that too.

*We could only identify two alignment-free methods, and their scope is rather limited.*

*One method only focuses on rearrangements (Smash 2015, six times cited) and one*

*method only on ALU insertions (AluMine 2019, cited 1 time). After careful consideration,*

*we have decided not to discuss alignment-free methods in this review.*

Reviewer #2:

This manuscript reviews different algorithms and approaches to SV calling (e.g. short vs. long

reads, DNA vs. RNA seq) and the benefits and limitations of each. It describes the general

difficulties in detecting SVs that are shared by all callers including inferring the SV type from the

alignment, standardizing the output of SV callers, and genotyping SVs across populations. This

is an important subject and the manuscript provides a good introduction to the benefits and

challenges of SV calling and a useful table that lists each caller with the type(s) of SV it best

detects and a link to download the code. However, the presentation could be clearer and more

complete.

*We thank the reviewer for their positive assessment and constructive feedback. We*

*incorporated many of the suggestions, and hope that this revised version is clearer and more*

*complete.*

Major Comments

1. Structural variants are difficult to understand. Since this is a review, I would add some

paragraphs towards the beginning describing different types of SVs and issues in calling them

(e.g. determining location, amplitude of copy-number change, phasing). This would deserve a

figure I think.

*We agree that SVs are difficult to understand and their complexity also often causes biases in*

*analyses. We start the introduction with the standard definitions of SVs according to multiple*

*publications. The main purpose of Figure 1 is to highlight the different types of SVs and how*

*short- and long-reads can help identify them.*

Currently, the description is that SVs are “genomic differences”—unacceptably vague.

*We have reworked the description of SV. However, we note that the reviewer’s excerpt is only*

*the beginning of the first sentence of the introduction. The whole first page of the introduction,*

*alongside Figure 1, are all devoted to describing SVs.*

Also, their description as &gt;50 bp is entirely arbitrary and not a consistent convention. It would

be reasonable to describe the range of alterations, and why they are split between indels, large

SVs, and the SVs in between (largely because different algorithms have been used to detect

indels vs large SVs, and the others have been a blind spot).

*We now mention that the 50bp threshold is arbitrary. It is however widely used (e.g. refs [1–6]).*

*We also included a discussion about size ranges and resolution of methods in the individual*

*sections about de-novo assembly-based mapping based for short and long read approaches.*

2. Indeed, I don’t think “phasing” is ever described in this review, despite its central importance

in understanding the consequences of an SV. The ability to phase SVs would be a major selling

point for any SV caller.

*We agree. Phasing is an emerging area, with currently very few methods that are able to phase*

*SVs directly. One method, which works for small to midsize deletion is WhatsHap. Another is*

*Crosstich, but it is still under development and is, as of yet, unpublished. We have extended the*

*discussion section to cover this.*

3. It is important to distinguish the specific challenges in calling somatic vs germline events and

vice versa. E.g. purity and the “somatic” call in the case of somatic alterations; what to consider

as the reference genome in the case of germline alterations. Alternatively, explicitly state

(including in the title) that this review is focused on germline events.

*Indeed this is an important consideration for cancer and disease-driven research. At a*

*methodological level, unfortunately, most often this distinction relies on question-specific*

*experimental design and additional ad-hoc filtering, and remains challenging. Few generic tools*

*have been devised for this problem. As a result, we chose not to focus on this aspect in our*

*review.*

4. The manuscript is focused on rearrangements and does not really mention their relation to

copy-number alterations (also considered SVs), or the challenges and approaches to detecting

telomeric alterations. For the latter, the manuscript should at least note that these exist and are

the subject of a different set of algorithms, which will not be discussed here.

*We agree with the reviewer that the relationship to copy number alterations could be made*

*more explicit. We have extended the introduction to address this point.*

5. The focus on single events in the first paragraph should be expanded on: complex

events have not only been reported on; they are common, especially (but not exclusively) in the

somatic realm. Often they result from a single “event” that generates multiple rearrangements;

this can be clarified. The manuscript should cite a chromothripsis paper here as well.

*This is an excellent point, as we did not mention chromothripsis in the previous version of our*

*review. We have extended the introduction to also cover this aspect. We did mention that*

*complex SVs exist in general citing two previous studies. We are however, not aware of studies*

*showing that more complex events are more common than standard SVs events.*

6. The importance of SVs should be highlighted more early in the introduction, rather than

burying it in the middle of a paragraph. After all, they account for 15X as much variation

between human genomes as SNVs (and something along these lines should be explicitly

stated).

*We highlight the importance of SV in the 4th sentence of the introduction, which to our mind is*

*not really “burying" it. However, we of course agree with the reviewer of the paramount*

*importance of SV and have added an entire new box (Box 2) on the impact of SVs on human*

*diseases and plant phenotypes.*

7. Box 1 doesn’t really describe the roles of SVs in medicine and biology (it really just

focuses on the challenges in calling SVs). A box that really did this would be useful.

*We have added this information highlighting a couple of examples of SVs impacting different*

*diseases in humans and their impact on interesting plant phenotypes in Box 2.*

8. “De novo” assembly needs to be explicitly distinguished from local assembly

approaches. The former include algorithms like DISCOVAR (which might be included in the

manuscript); the latter include algorithms like MANTA (which is incorrectly classified as a

mapping algorithm), novobreak (which is incorrectly classified as de novo assembly) and Svaba

(which might also be included in the manuscript). Indeed, I would add local assembly

algorithms as a separate section to the existing sections on de novo and mapping-based

algorithms. It mediates between the two.

*The distinction between “de novo” and mapping-based is not as clear cut as one might think.*

*For instance, while MANTA indeed performs local de novo assembly, it mainly relies on*

*mapping to a reference genome. That being said, we followed the reviewer’s suggestion of*

*adding Svaba.*

9. In the paragraph indicating that SVs have been understudied, perhaps the main reason

has been left out: they largely require whole genome sequencing (and why this is more the case

for SVs than SNVs can be explained). Also, that beyond difficulties in calling them, they can be

difficult to understand because each SV can cover a large region of the genome and have longrange effects.

*While this argument makes sense we did not find a reference for it. Thus, we did not mention it*

*in the introduction as a potential cause.*

10. The description of long-range sequencing technologies should include 10X sequencing.

This is becoming widely used and is conceptually different from PacBio/Nanopore.

*We have now created a new Box 3 reviewing these technologies and methods.*

11. In describing individual algorithms, it would be useful to indicate whether they work on

somatic genomes, germline genomes, or both. This might be a column for the algorithms table,

for instance.

*Unfortunately, not many methods do distinguish between these two variant classes for SVs.*

*Apart from Delly, Manta and SV-Bay there are no other Structural Variant detection tool that*

*state clearly if they identify germline or somatic variants.*

12. BRASS and dRanger/Breakpointer might be included as additional short-read alignment

algorithms. It would also be useful to refer to methods that integrate other datasets, such as

Dixon et al Nat Gen 2018, who integrated HiC and optical mapping data to detect SVs.

*BRASS and Breakpointer were both designed to only detect rearrangements, and not the full*

*spectrum of SVs. Thus, we did not include them since we have already included multiple*

*methods that can identify all SVs types.*

13. I might include less description of individual methods in the text; the details could be left

to tables or supplementary tables describing the different methods. The text should instead be

used to make the overarching themes clearer, including the issues I described above.

*We have tried to incorporate some of the themes suggested by the reviewer, and stress that not*

*all methods included in the table are described in the main text. However, we feel that*

*combining high-level conceptual ideas with selected methodological details make for a more*

*didactic and engaging review.*

Minor Points:

1. In the long reads mapping section, the following run-on sentence should be subdivided.

*“Sniffles operates on a per read base capable of reporting of reporting also very low frequency*

*SVs in the sample, which is useful in cancer or for mosaic variation.”*

*We modified the sentence to ” Sniffles operates on a per read base capable of reporting also*

*very low frequency SVs in the sample. This is useful in cancer or for mosaic variation. “*

2. The axes of the plot in Figure 2a do not have a scale

*Indeed, Figure 2a conveys general trends among different approaches based on multiple*

*studies, and is more qualitative than quantitative. We now mention this in the caption.*

3. The caption in Figure 2b uses both word and numeric representations of numbers. It

should consistently signify numbers through words (e.g. two) or numerals (e.g. 2).

*We changed this to :” The ratio of improvement in number of SVs detected from using long*

*reads for four human and two non-human studies. Overall, each study shows a clear*

*improvement of using the longer reads. Supplementary Table 1 shows the details of each*

*study. “*

4. There should be a heading for the Table after Figure 2

*We have moved the table caption, which was originally at the end of the table, to the top.*

*References:*

*1. Devi KS, Sumangala Devi K, Department of Anatomy, SVS Medical College,*

*Mahaboobnagar, Telangana, et al. STUDY OF ABNORMAL LOBAR PATTERN OF LUNGS*

*[Internet]. International Journal of Anatomy and Research. 2016. p. 2251–7. Available from:*

*http://dx.doi.org/10.16965/ijar.2016.190*

*2. Sudmant PH, Rausch T, Gardner EJ, Handsaker RE, Abyzov A, Huddleston J, et al. An*

*integrated map of structural variation in 2,504 human genomes. Nature. 2015;526:75–81.*

*3. Sedlazeck FJ, Rescheneder P, Smolka M, Fang H, Nattestad M, von Haeseler A, et al.*

*Accurate detection of complex structural variations using single-molecule sequencing. Nat*

*Methods. 2018;15:461–8.*

*4. Sedlazeck FJ, Lee H, Darby CA, Schatz MC. Piercing the dark matter: bioinformatics of longrange sequencing and mapping. Nat Rev Genet. 2018;19:329–46.*

*5. Gudbjartsson DF, Helgason H, Gudjonsson SA, Zink F, Oddson A, Gylfason A, et al. Largescale whole-genome sequencing of the Icelandic population. Nat Genet. 2015;47:435–44.*

*6. Chaisson MJP, Sanders AD, Zhao X, Malhotra A, Porubsky D, Rausch T, et al. Multi-platform*

*discovery of haplotype-resolved structural variation in human genomes. Nat Commun.*

*2019;10:1784.*

*7. Nagarajan N, Pop M. Sequence assembly demystified. Nat Rev Genet. 2013;14:157–67.*
